# Electrochemical Performance of Fe40Al-X (X = Cr, Ti, Co, Ni) Alloys Exposed to Artificial Saliva

**DOI:** 10.3390/ma13051095

**Published:** 2020-03-01

**Authors:** Cinthya Dinorah Arrieta-Gonzalez, Roberto Ademar Rodriguez-Diaz, Jan Mayen, Rogel Fernando Retes-Mantilla, María Teresa Torres-Mancera, Lya Adlih Oros-Méndez, Héctor Cruz-Mejía, Nestor Starlin Flores-Garcia, Jesús Porcayo-Calderon

**Affiliations:** 1 Tecnológico Nacional de Mexico–Instituto Tecnológico de Zacatepec, Calzada Instituto Tecnológico 27, 62780 Zacatepec, MOR, Mexico; cdaglez@gmail.com; 2 Centro de Cooperación Academia–Industria, Tecnológico Nacional de Mexico, Tecnológico de Estudios Superiores de Coacalco, Av. 16 de septiembre 54, Col. Cabecera municipal, 55700 Coacalco de Berriozábal, EDO. MEX., Mexico; rdiaz.unam@gmail.com (R.A.R.-D.); retes@tesco.edu.mx (R.F.R.-M.); teresa@tesco.edu.mx (M.T.T.-M.); 3 CONACYT-CIATEQ, Unidad San Luis Potosí, Eje 126 No. 225, Zona Industrial, 78395 San Luis Potosí, S.L.P., Mexico; dr.jmayen@gmail.com; 4 División de Ingeniería Industrial, Tecnológico Nacional de Mexico, Instituto Tecnológico Superior de San Luis Potosí, Capital, Carretera 57 Mexico-Piedras Negras km 180+100 No. 6501. Delegación Villa Pozos, 78421 San Luis Potosí, S.L.P., Mexico; lya.oros@tecsuperiorslp.edu.mx; 5 División de Ingeniería en Nanotecnología, Universidad Politécnica del Valle de Mexico, Av. Mexiquense s/n, 54910 Tultitlán, EDO. MEX., Mexico; hcruzmejia@gmail.com; 6 Instituto de Ciencias Físicas, Universidad Nacional Autónoma de Mexico, Avenida Universidad s/n, 62210 Cuernavaca, MOR, Mexico; nestor@icf.unam.mx; 7 CIICAp, Universidad Autónoma del Estado de Morelos, Avenida Universidad 1001, 62209 Cuernavaca, MOR, Mexico

**Keywords:** intermetallics, artificial saliva, electrochemical technics, corrosion, biomaterials

## Abstract

Fe–Al intermetallic compounds have been considered excellent candidates as alternative alloys for various applications in corrosive environments compared to other Fe-based alloys. Their excellent corrosion resistance is due to the development of an Al-based passive layer. The performance of the passive layer can be improved by adding a third alloy element. Therefore, in this study the electrochemical performance of the Fe40Al intermetallic alloy modified by the addition of a third alloy element (Cr, Ti, Co, Ni) is evaluated. The corrosion resistance of intermetallic alloys has been evaluated by electrochemical tests (potentiodynamic polarization curves, and measurements of open circuit potential, linear polarization and electrochemical impedance) in artificial saliva. The performance of intermetallic alloys was compared with that of Ti. The results obtained showed that the addition of Ni and Ti substantially improves the corrosion resistance of the base intermetallic. The corrosion resistance shown is comparable or greater than that shown by Ti. However, the addition of Co reduces the corrosion resistance of the base intermetallic.

## 1. Introduction

A biomaterial is developed in order to be utilized in physical contact with live tissue; for that reason, it is important that the implanted material will not produce dangerous effects in the human body. Thus, non-toxic metallic implants must be fabricated in such way that they will not induce allergic reactions or inflammation in the human body. Biocompatibility is the capability of a biomaterial to co-exist with human body tissues without inducing significant damage to the body system. This property is associated not only to toxicity but also to all the possible effects of a material in the biological system [1,2,3].

The choice of biocompatible metals is a relevant issue in corrosion resistance as the chemical degradation of metallic implants when exposed to biological fluids cannot be avoided [4]. Implants release undesirable metal ions which are non-biocompatible. Corrosion can induce a reduction of the lifetime of the implanted biomaterial and thus, a second surgery will be required. A metal that behaves passively or inertly in the air can display a great corrosion rate in the human body. A metallic implant that is intended to be used inside the oral cavity environment must possess high corrosion resistance and good biocompatibility.

Corrosion of dental implants of metallic nature can induce adverse effects in the biological human system by affecting its functionality and also can modify the esthetic of natural or prosthetic dental pieces. The metallic ions and by-products generated during electrochemical corrosion process can induce detrimental effects on tissues and cell in the surrounding biological media. In the same way, these corrosion products can be transported by biological fluids to different parts of the human body. In this sense, the biomedical use of dental alloys in the oral cavity requires an extensive study of its corrosion performance [5,6].

It is well known that iron aluminides possess excellent oxidation and corrosion performance, and they are considered as light compounds compared to other Fe-based alloys [7,8]. The Al content of these aluminides promotes the formation of a passive layer of aluminum oxide, which is responsible for the good oxidation, corrosion, and sulfidation resistance at room temperature or higher [9]. In addition, these intermetallic compounds preserve a good mechanical strength and stiffness at high temperatures [10,11].

During the last decades, iron aluminide compounds have been the subject of considerable interest focused on their aqueous corrosion properties. Their corrosion performance has been investigated in a wide range of electrolytes such as acidic, basic, chloride and sulfur compound solutions [12,13,14,15,16]. Although iron aluminides have been studied mainly for structural applications at elevated temperature due to its capability to form a passive layer of Al-oxide that induces a good corrosion resistance even in molten salt environments [17,18], its excellent behavior in these conditions has motivated its study in aqueous solutions. Previous investigations have established that the iron aluminides can be considered as potential biomaterials in room temperature applications [19,20,21]. It is important to remark that the corrosion performance of iron aluminides have not been evaluated when exposed to artificial saliva; however, some studies related to the corrosion evaluation of this kind of intermetallics when exposed to other biomimetic media or chloride containing solutions have been developed.

Garcia-Alonso et al [22] studied the corrosion performance of an Fe_3_Al-base intermetallic compound which presented different crystal structures in a Hank solution. The results of this research showed that the crystal structure of intermetallics did not affect significantly the corrosion rate expressed in terms of the corrosion current density, besides; these materials exhibited a stable passive state during the whole test. These corrosion rates of the Fe_3_Al-base intermetallics were of the same order of magnitude as for 316L stainless steel.

Castañeda et al [23] studied the corrosion resistance of Ni–Al–Fe intermetallic alloys in biomimetic human body fluid electrolytes. The tested alloys included 57 (wt%) Ni- (20 to 30) Al- (12 to 23) Fe and they were exposed to the Hank’s solution. The corrosion performance of these ternary Ni–Al–Fe intermetallics was compared with that of the AISI 316L type stainless steel. The corrosion tests have shown that these alloys exhibited a similar or higher corrosion resistance than the AISI 316L type stainless steel, and this corrosion resistance decreased as the Al concentration in the intermetallic alloy was augmented. The ternary intermetallic alloys were susceptible to pitting-type corrosion on the interdendritic Ni-rich phases.

Arrieta-Gonzalez et al [20] evaluated the corrosion resistance of Fe_3_Al-type intermetallic alloys when they were exposed in Hank´s solution. Different concentrations of Ni were added to the Fe_3_Al intermetallic alloy and they were tested in the as-cast and thermal-treated conditions. For comparison, the corrosion performance of Ti and 316L stainless steel was also assessed. The results derived from this research have shown that the 316-L stainless steel and Ti are the materials with greater corrosion resistance in chloride-rich environments. Compared to 316-L SS and Titanium, intermetallic Fe_3_Al alloys had greater susceptibility to pitting corrosion. Ni addition and thermal treatment induced a beneficial effect on the corrosion resistance of Fe_3_Al-base alloy owing to an improved stability of the passive layer.

Sarmiento Bustos et al. [19] studied the effect of the addition of Ag on the corrosion behavior of the iron aluminide Fe40Al (at. %) in a biomimetic environment that simulates the physiological human body electrolyte. In this research, the corrosion rate of Fe40Al2.5Ag alloy turned out to be lower than that of binary Fe40Al alloy; however, according to the linear polarization resistance test, the current density of the ternary alloy was higher than that of the binary FeAl-based alloy. This behavior is due to the enhancement of the protective nature of the Al oxide layer of the Fe40Al aluminide, while the immersion time in the Hanks´ solution had advanced.

Due to the excellent corrosion resistance properties that Fe-Al intermetallic alloys have shown, it is of interest to explore their performance in simulated biological fluids in order to determine their behavior as a possible biomaterial. In addition, it is important to determine whether the addition of a third alloy element such as Ti, Cr, Co and Ni contributes to an improvement in the electrochemical behavior of the base alloy against the corrosive action of the electrolyte used. Therefore, the objective of this research work is to carry out an evaluation of the electrochemical behavior of the Fe40Al intermetallic alloy modified with the addition of Ti, Ni, Co, and Cr. The electrochemical techniques used were polarization curves and measurements of open circuit potential, polarization resistance, and electrochemical impedance in synthetic saliva.

## 2. Materials and Methods 

### 2.1. Materials

Cast ingots of ternary Fe40Al-3X (X = Cr, Ti, Co, Ni) (weight %) alloys were elaborated by utilizing an induction furnace at about 1600 °C in air. Fe, Al, Cr, Ti, Co, and Ni of high purity (99.9%) were collocated inside a SiC crucible in order to be melted. The casting process of the Fe40Al based alloys were carried out by pouring the molten metal into a rectangular steel mold, then the alloys underwent the solidification process during cooling until room temperature was reached. The ingots fabricated in this way showed a coarse-grain microstructure. X-ray diffractometry (XRD, Rigaku Dmax2100 with a Cu-Kα radiation, (Tokyo, Japan) was used to characterize the alloys.

### 2.2. Artificial Saliva

Fe40Al based alloys were immersed in the biomimetic solution. In this case, the artificial saliva electrolyte was utilized as the corrosion environment, and the concentration of the constituents of the artificial saliva is shown in Table 1 [24].

### 2.3. Electrochemical Corrosion Tests

Electrochemical corrosion experiments were performed by utilizing an ACM Instruments zero-resistance ammeter (ZRA) (potentiodynamic polarization measurements) and an Interface 1000 Gamry Potentiostat/Galvanostat/ZRA (Gamry Instruments, Warminster, PA, USA), both coupled to a personal computer.

The electrochemical cell used was a typical three-electrode one, whereas the reference electrode (RE) was a saturated calomel electrode (SCE), and the counter electrode (CE) a Pt wire. Fe40Al and ternary alloys were the working electrodes (WE).

The work electrodes were manufactured by encapsulating the alloys (with dimensions, 1 × 1 × 0.3 cm) in epoxy resin in acrylic molds. Previously a copper conductor wire was welded to the metal samples by the spot-welding technique. For corrosion tests, the encapsulated samples were abraded with sandpaper grade 120 to 600, subsequently washed with distilled water and ethanol and immediately immersed into corrosive electrolyte.

Corrosion resistance of the alloys was determined by potentiodynamic polarization from −400 mV to 1000 mV with respect to corrosion potential (Ecorr). The polarization scans were carried out at a rate of 1 mV/s. Prior to the electrochemical evaluation, the prepared specimens were left to stabilize for 20 min. Electrochemical corrosion parameters (corrosion current density (icorr), icorr, Tafel slopes, Ecorr) were determined by utilizing the extrapolation Tafel method from ± 250 mV around the corrosion potential (Ecorr).

The electrochemical behavior of the alloy-electrolyte interface will be characterized by electrochemical impedance spectroscopy (EIS, Gamry Instruments, Warminster, PA, USA). The analysis of the Nyquist and Bode diagrams will determine the presence of both the Faradaic and non-Faradaic processes that influence the degradation-protection process of the alloys evaluated. The impedance spectra obtained by the electrochemical impedance spectroscopy technique were recorded at the open circuit potential (OCP), which was executed in the frequency range of 0.01 Hz to 100,000 Hz with a perturbation of ±10 mV.

## 3. Results and Discussion

### 3.1. Microstructural Characterization

**Figure 1:**
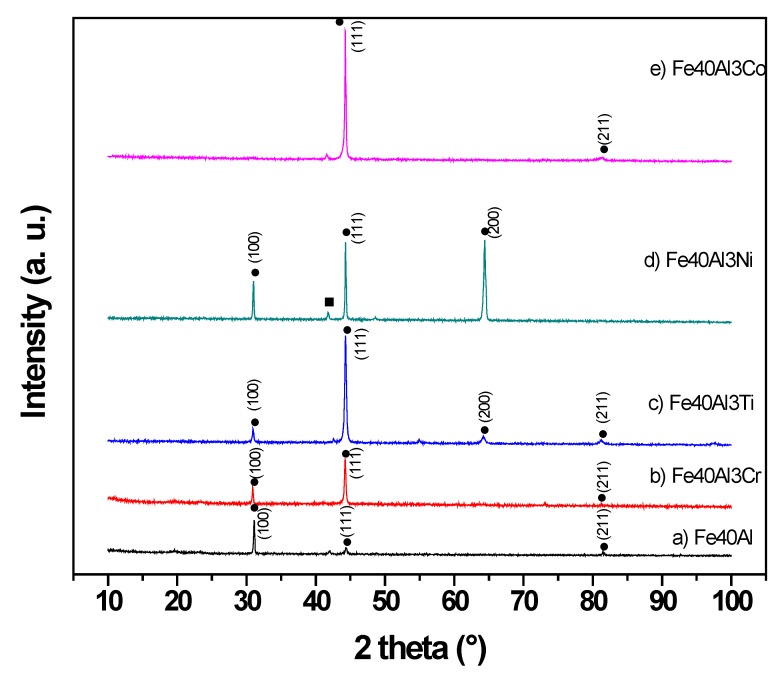
X-ray diffraction profiles of the as-cast intermetallic alloys (**a**) Fe40Al, (**b**) Fe40Al-3Cr, (**c**) Fe40Al-3Ti, (**d**) Fe40Al-3Ni and (**e**) Fe40Al-3Co.

Figure 1 displays the X-ray diffraction spectra of as-cast Fe40Al and ternary Fe40Al-3X (weight %) (X = Cr, Ti, Ni and Co) alloys. From this illustration, it can be observed that all the ternary alloys exhibited between two and four diffraction peaks within the range of 2ϴ studied. The absence of some diffraction peaks in all the X-ray profiles is associated with a coarse grain size of the order of microns to millimeters. As is well known, when these types of intermetallic are melted with a subsequent casting process in a metal mold, then the resulting microstructure is composed of coarse grains. The indexation of all diffraction patterns revealed an ordered crystal structure type B2 that corresponds to the FeAl phase. This finding indicates that the addition of alloying elements did not alter the crystal structure of the binary intermetallic alloy. Also, the variation of intensity of the (111) diffraction peak corresponding to the binary Fe40Al intermetallic alloy is associated with a preferred crystallographic orientation. Regarding to the Fe40Al-3Cr alloy, its X-ray diffraction pattern revealed that the Cr element entered in solid solution in the binary Fe40Al matrix, which is related to the absence of peaks different to that of the binary intermetallic phase, and this finding is in agreement with the ternary alloy phase diagram of the system Fe-Al-Cr [25]. Previous studies have reported that all binary Fe-Al intermetallic phases exhibit significant solubility for Cr. This might be attributed to the very similar atomic radii of Fe and Cr.

### 3.2. Polarization Curves

**Figure 2:**
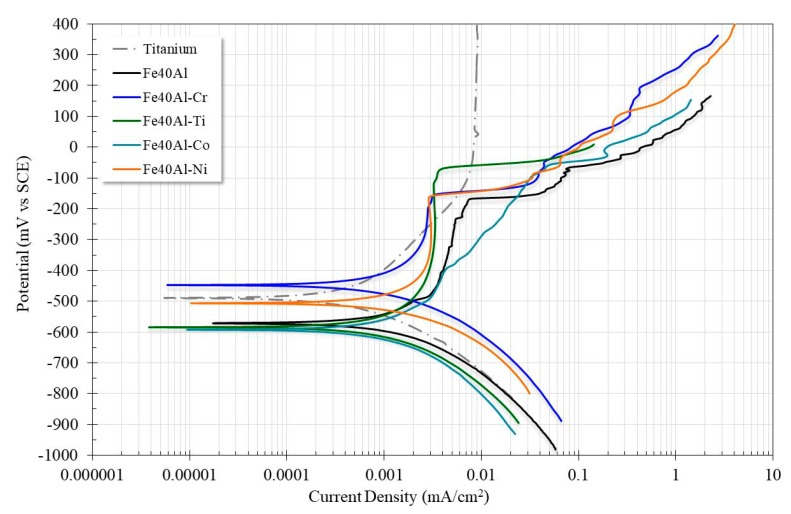
Potentiodynamic polarization curves of the alloys exposed to artificial saliva.

Figure 2 shows the potentiodynamic polarization curves of the Fe40Al based alloys exposed to the synthetic saliva electrolyte. It can be observed from this graph that all the ternary Fe40Al-X alloys exhibited a dual active-passive behavior. However, Ti exhibited a high stability in the synthetic saliva, since this material showed a passive behavior in the biggest interval of potential with respect to it corrosion potential. Considering the group of Fe40Al based alloys, the ternary Fe40Al-3Co alloy showed the more active corrosion potential, while the Fe40Al-3Cr alloy exhibited the nobler Ecorr. With respect to the corrosion rate represented in terms of the corrosion current density, icorr, it can be said that the addition of Cr, Ni, Co and Ti to the Fe40Al base alloy induced a decrease of its corrosion rate, where, the addition of Ti was the element that enhanced more favorably the corrosion resistance of the binary alloy. It is worth noting that the Fe40Al-3Ti alloy exhibited an icorr approximately equal to that of Ti. The results reported here agree with several studies that report that the addition of third alloy elements improves the corrosion resistance of iron aluminide [22,26,27,28,29].

Even though the corrosion properties of the Fe40Al alloy were enhanced significantly after the addition of Cr, Ni, Ti and Co, all these ternary alloys remain susceptible to pitting corrosion, see Table 2. This behavior can possibly be attributed to the high content of Al in the binary Fe40Al based alloy. The anodic polarization curve of the binary alloy exhibits a passive zone within an interval of potential of 400 mV above its corrosion potential; beyond this overpotential, the passive layer fails and the material start to be susceptible to pitting corrosion. Also, Table 2 shows that the passivation and pitting process of Fe40Al-3Co alloy are not clearly defined. Ti exhibited the more stable passive behavior during the whole interval of potential. It is worth noticing that Fe40Al-3Ti alloy exhibited the wider interval of potential that delimited the passivation regime before pitting process had begun. In contrast, Fe40Al-3Cr alloy showed the lower interval of potential that delimited the passivation zone.

The corrosion rate (mm/yr) of the alloys evaluated was calculated (Table 2). From the results all the Fe40Al-X alloys showed corrosion rates lower than that shown by the binary alloy (Fe40Al). However, the Fe40Al-3Ti alloy exhibited a corrosion rate minor than the other ternary alloys and very similar to that shown by Ti. This behavior could be associated to the modification of the Al-oxide by TiO_2_. Since it is well known than the protective film on titanium dental implants is constituted predominantly of titanium oxide (TiO_2_), with a rutile-type tetragonal structure [30,31].

Since recent decades, intermetallic compounds based on aluminides have been considered as candidate materials to be used as biomaterials; in this sense, is important to compare the corrosion rate of alloys studied in present work with other intermetallic compounds when they were exposed to artificial biomimetic media. For example, Castañeda et al. [23] studied the corrosion behavior of a group of Ni-Al-Fe intermetallic alloys when they were exposed to Hank´s solution; in this case the Al concentration ranged from 20 to 30 wt.% and the Fe concentration ranged from 13 to 23 wt.%. It is worth noticing that all the ternary Fe40Al-3X alloys studied in this research, presented minor corrosion rates than those of the ternary Ni-Al-Fe intermetallics, since the corrosion current density of these alloys ranged from 0.46 to 1.2 mA/cm^2^. In this sense, the best corrosion rate of Fe40Al-3X alloys as compared with that of ternary alloys is certainly due to the major Al content contained in the former alloys, which promoted the formation of a more uniform and ticker Al-oxide scale on the surface of aluminides. In a similar research, Arrieta-Gonzalez et al. [20] evaluated the corrosion performance of binary Fe_3_Al and ternary Fe_3_Al-X (X = 1, 3, 5 Ni wt.%). The authors reported corrosion current densities of 0.00095, 0.0065, 0.002 and 0.0028 for Fe_3_Al, Fe_3_Al-1Ni, Fe_3_Al-3Ni, and Fe_3_Al-5Ni respectively. In this case, the Fe40Al-3Cr, Fe40Al-3Ti, Fe40Al-3Co, and Fe40Al-3Ni studied in present research presented lower corrosion rates than those of ternary Fe_3_Al-based alloys. Similarly, this behavior can be ascribed to the higher Al concentration contained in the Fe40Al based intermetallic alloys, which in turn give place to the formation of a more uniform and ticker layer onto the surface of aluminides.

Table 3 displays a compilation of corrosion rates of selected noble and Ti-, Ni-, and Zr-based alloys. In vitro corrosion tests of these metallic biomaterials exposed to artificial saliva where developed at room temperature. In this case, the corrosion rates of Fe40Al, Fe40Al-3Cr, Fe40Al-3Co, and Fe40Al-3Ni were lower than those of the Ney-Oro-B2 (75.7Au-4.7Pd–11.1Ag–8.4Cu–1.4Zn) [31] noble alloy, Vera Bond (76.9Ni-12.6Cr-5Mo-2.9Al-2Be), and Bio Bond (80.7Ni-13.3Cr-4V-1.8Be) [32]. The fact that the mentioned Fe40Al-X alloys showed lower corrosion rates than that of the high noble alloy (Ney-Oro-B2) could be associated to the highly protective Al-oxide scale developed on surfaces of iron aluminide compounds, although the standard electrode potential of the Au-based alloys is nobler. Similarly, the binary Fe40Al and the ternary Fe40Al-X alloys with additions of Cr, Co, and Ni exhibited lower corrosion rates than those of Ni-based alloys (Vera Bond and Bio Bond). This behavior could be explained in terms of the protective nature of the oxide films developed on the tested alloys, in this case, the aluminum oxide layer formed on the surface of iron aluminides is more protective than the Cr and Mo oxides, which are responsible of the corrosion resistance of these Ni-based alloys. Also, it is worth noticing that the Fe40Al-3Ti alloy shows a similar degradation rate to the Ti6Al4V commercial alloy, as reported in a previous research by Kuphasuk [33]. This behavior is certainly attributed to the favorable effect derived of the modification of the protective nature of the Al-oxide scale by the TiO_2_ oxide formed onto the surface of the ternary Fe40Al-3Ti aluminide.

Table 2 and Table 3 exhibit that corrosion rate of Fe40Al-3Ti alloy evaluated in the present work was lower than those of Ni-Cr-Mo-Al-Be, Ni-Cr-V-Be, Ney-Oro-B2, and most Zr-Ti dental alloys.

### 3.3. Open Circuit Potential

**Figure 3:**
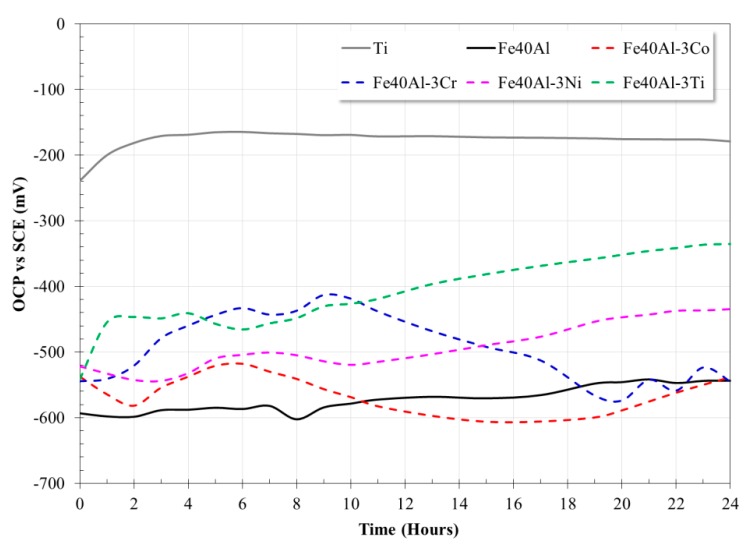
Variation of open circuit potential (OCP) of the evaluated alloys as a function of exposure.

Figure 3 shows the dependence of the OCP as a function of immersion time of the evaluated alloys. The variation in the OCP values as a function of time, allows us to determine the type of interaction of the alloy with the electrolyte. In general, an increase in the OCP values is an indication of the formation of a passive film onto alloy surface; a trend without changes in the OCP values indicates that the alloy surface is not undergoing a corrosion process; however, a decrease in the OCP values is indicative of an active corrosion process and the inability of the alloy to develop a protective film that protects it. Ti exhibits a significant transient behavior in the anodic direction during the first three h in contact with the biomimetic electrolyte. After this period, the open circuit potential of Ti remained more or less constant. The stable behavior after 3 h of exposure could be associated with the stable Ti-oxide protective film formed on its surface. Figure 3 also shows that the addition of Ni to Fe40Al alloy promoted a shift of OCP values towards the nobler direction during most of exposure time; it became nobler as the immersion time evolved. This behavior has been observed in previous investigations [20] where it was observed that the addition of Ni to Fe_3_Al alloy caused a shift in its OCP values towards more noble values; in addition, the addition of this element improved its corrosion resistance due to an increase in the passive layer’s stability. Also, the intermetallic alloy modified with the addition of Ti showed a similar behavior: it showed nobler OCP values. These behaviors could be associated with the improvement of the protective nature of the oxide scale formed on surface of these ternary intermetallics and this in turn could have been related to the modification of the aluminum oxide layer. Addition of Cr induced a shift of the OCP values towards the noble side during the first 15 h of immersion; however, after that, the OCP values became more active than the binary alloy. A similar behavior was observed with the addition of Co. In both cases, after 20 h of immersion, their OCP values were similar to those of the binary alloy.

### 3.4. Linear Polarization Resistance (LPR) Measurements 

**Figure 4:**
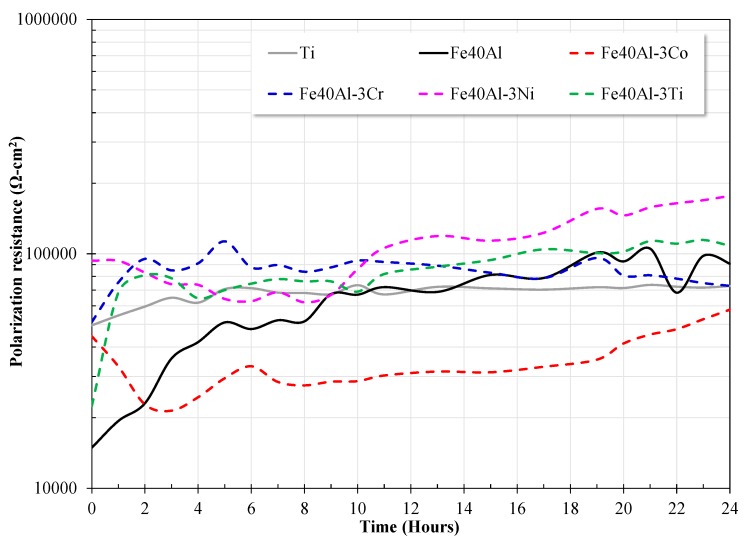
Variation of polarization resistance (Rp) of the evaluated alloys as a function of exposure period in artificial saliva.

The fluctuation of Rp as a function of immersion period for the evaluated alloys is showed in Figure 4. The Rp values of Fe40Al-3Co, Fe40Al-3Cr, Fe40Al-3Ni alloys showed a fluctuating behavior while the exposure time advanced. However, Rp values of all alloys increased from the beginning of electrochemical tests up to 24 h in contact with the synthetic saliva electrolyte. Also, during the whole exposure time, the Fe40Al alloy modified with Ni presented the higher Rp values as compared with those of binary Fe40Al base alloy. Besides, after 10 h of immersion, Fe40Al-3Ni alloy, exhibited the highest Rp values of all intermetallic alloys, even higher than Ti. The excellent performance of the intermetallic alloy modified with Ni addition could be attributed to the improvement of the adhesion of the external protective film by preventing the formation of voids or by reducing the quantity of precipitates [21]. Addition of Cr to Fe40Al base alloy induced a shift of Rp towards higher values during the first 15 h of immersion, however after this period; the Rp values of Fe40Al-3Cr displayed similar Rp values as compared with binary intermetallic alloy. The intermetallic alloy modified with the addition of Co showed the lowest performance. In the first three hours of immersion, it experienced a significant decrease in its Rp values, and subsequently showed a tendency to increase its corrosion resistance until reaching values close to those of the base alloy. It is observed that the Rp values of the Fe40Al and Ti alloy tended to converge at values not significantly different from each other at immersion times greater than eight hours. Also, Rp values of Fe40Al-3Ti prevailed higher than those of Fe40Al and Ti. This behavior may be due to the formation of a mixture of Al_2_O_3_ and TiO_2_ compounds on the surface of iron aluminide, as reported previously [38]. Also, the establishment of a protective layer composed of Al and Ti oxides is congruent to the fact that the formations enthalpies of Al_2_O_3_ and TiO_2_ are approximately close each other.

### 3.5. Electrochemical Impedance Spectroscopy Measurements 

**Figure 5:**
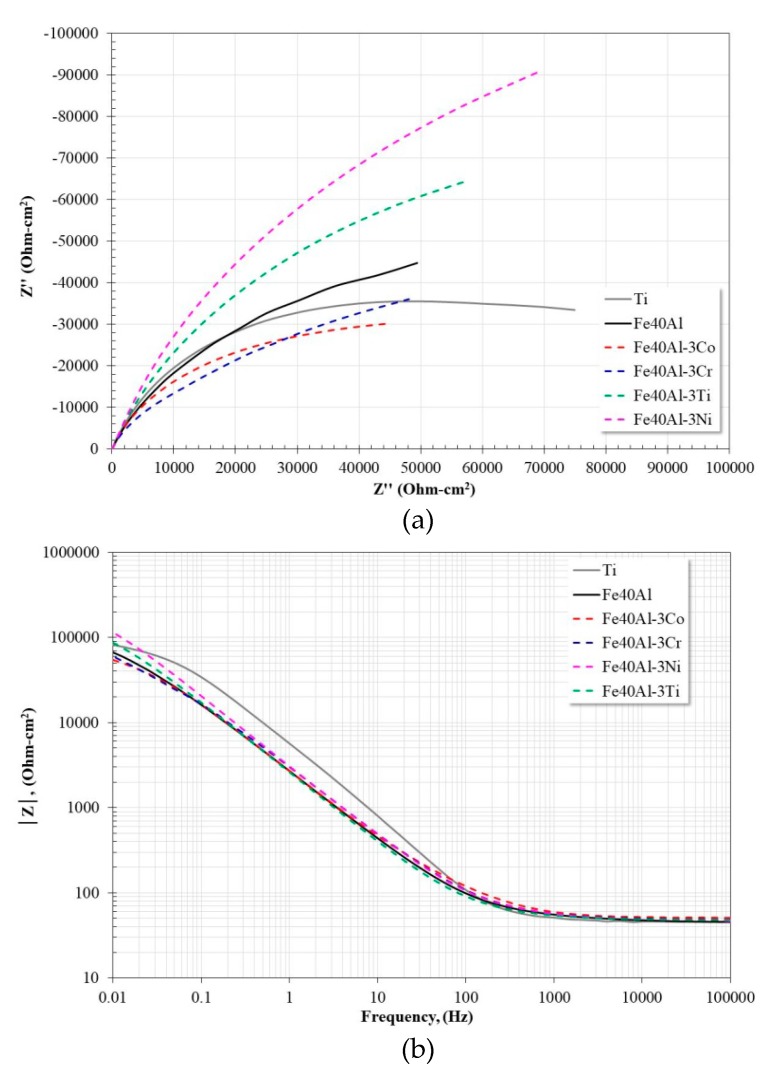

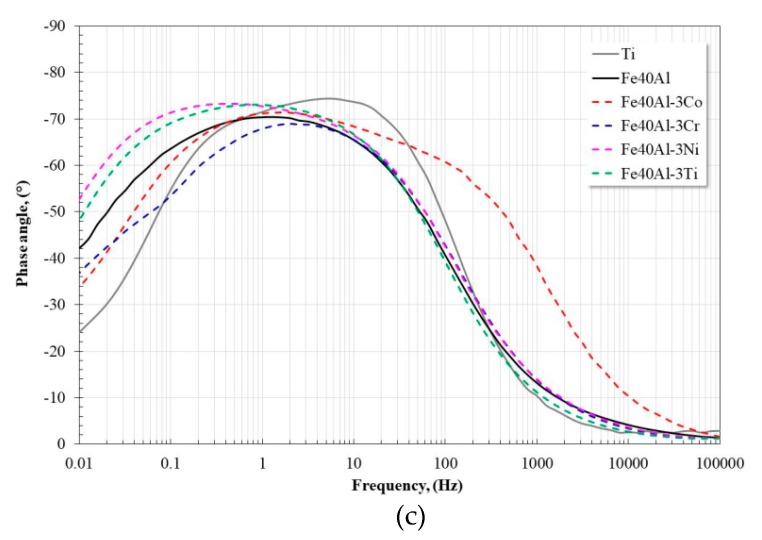
Nyquist (**a**) and Bode (**b**,**c**) plots for alloys exposed in artificial saliva at 37 °C after 24 h.

Figure 5 shows the impedance spectra of the materials evaluated in artificial saliva after 24 h of immersion. From the Nyquist diagram (Figure 5a) it is observed that all materials show the apparent development of a single capacitive loop. However, the observed plot only represents the experimental points of the low frequency region, which may correspond to the capacitive response of the metal surface. That is why the interpretation of the Bode diagrams provides more information about the corrosion-protection process; this is because all the information of the analyzed frequency range is observed in them. According to Figure 5b, in the high frequency region (> 1000 Hz) the development of the plateau that represents the resistance of the electrolyte is observed, and at intermediate frequencies (10< f <1000, Hz) the development of the linear relationship between the frequency and the impedance module is observed (log *f*-log |*Z*|). In general, it is observed that this relationship extends to the low frequency region, thereby indicating that the impedance module is greater than the last recorded value. The variation in the slope of the linear relationship is directly related to the variation of the maximum phase angle (Figure 5c), so that an increase in its slope implies an increase in its maximum phase angle and, therefore, an increase in the capacitive response of the metal surface. From Figure 5c, in the high frequency region, it is observed that for most materials, the phase angle tends to zero at frequencies greater than 1000 Hz, and, in the case of the intermetallic alloy modified with Co, its phase angle tends to zero at frequencies greater than 10,000 Hz. The above suggests the presence of corrosion products of the hydroxides- or oxyhydroxides-type adsorbed onto metal surface. The phase angle at lower frequencies (<1000 Hz) shows the presence of two time constants in all cases. The first one around 10 Hz (except the intermetallic modified with Co, 100 Hz) whose presence may be associated with the capacitive response of the metal surface, and the second one at frequencies below 1 Hz whose response may be associated with the capacitive response of the electrochemical double layer. In all cases, the phase angle tends to zero at frequencies below 0.01 Hz, which justifies the absence of the low-frequency plateau.

This analysis shows very similar results to those observed in the section of polarization curves and LPR measurements, that is, the modification of the Fe40Al intermetallic with the addition of either Ni or Ti substantially improved its corrosion resistance, showing values of charge transfer resistance, greater or similar to those shown by Ti. Because the evolution of the impedance spectra of ternary intermetallics is similar, for simplicity, the subsequent analyzes will be carried out on Fe40Al and Fe40Al-3Ni intermetallics, and Ti.

**Figure 6:**
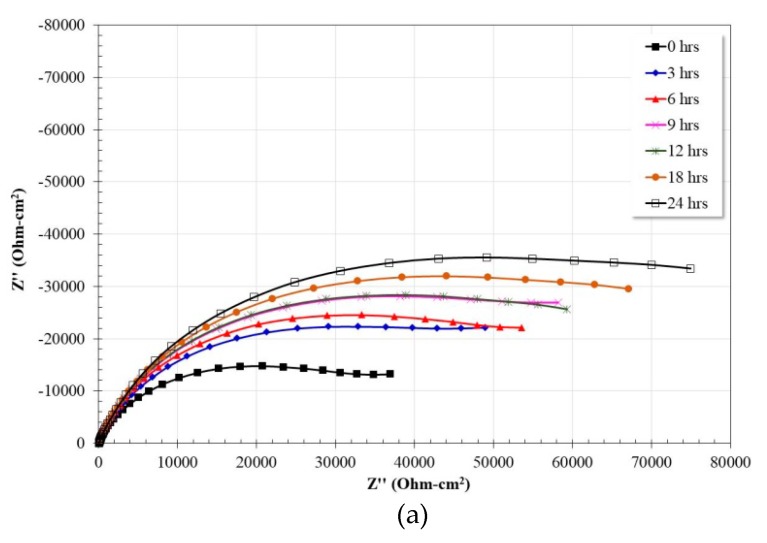

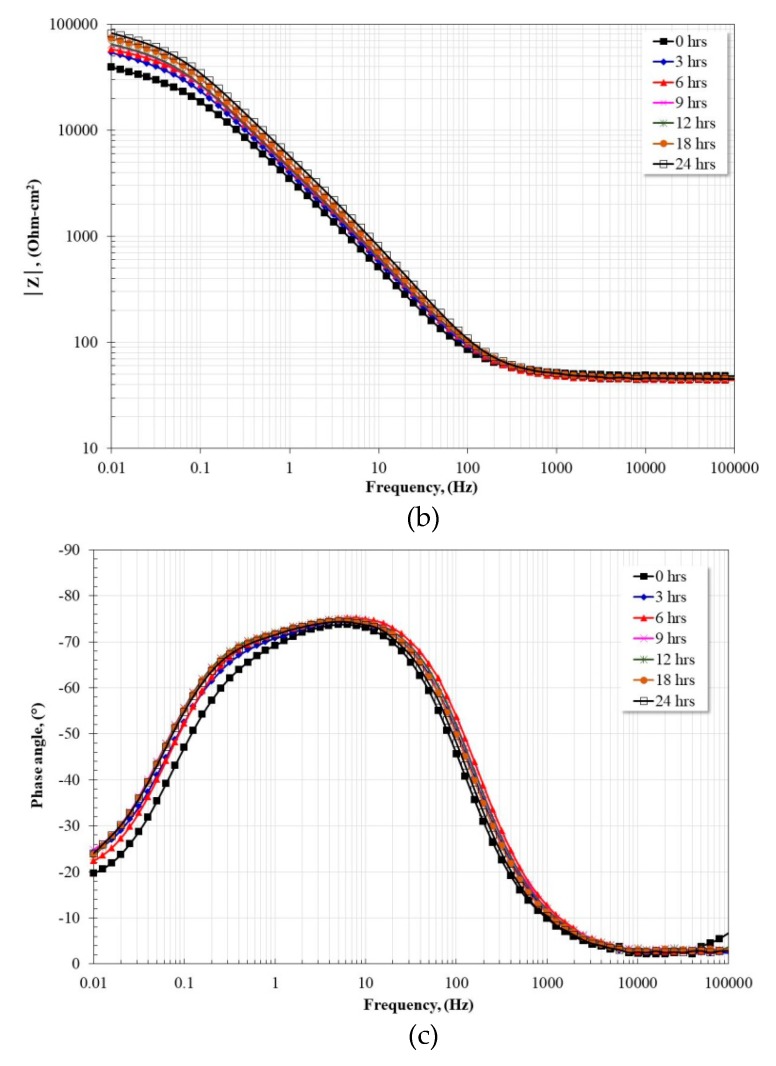
Nyquist (**a**) and Bode (**b**,**c**) plots for Ti exposed in artificial saliva at 37 °C after 24 h.

Figure 6 shows the variation of the impedance spectra of the Ti as a function of immersion time. From the Nyquist diagram (Figure 6a) the apparent presence of a single capacitive semicircle is observed, the diameter of which tends to increase as the immersion time elapses. This suggests a constant increase in the charge transfer resistance due to the formation and growth of a Ti-based protective oxide. On the other hand, in the high frequency region the presence of the corresponding plateau is observed (Figure 6b), which coincides with a phase angle tending to zero (Figure 6c). In the intermediate frequency region a linear relationship between the frequency and the impedance module with apparently a single slope is visible, however, according to the phase angle (in the same region) the presence of two maxima with similar values is observed (around 75° the first, and 70° the second). Therefore, in the impedance module graph the linear relationship apparently shows only one slope. At frequencies below 0.1 Hz, a decrease in the slope of the linear relationship is observed, however it is not possible to observe the formation of the low frequency plateau. Therefore, the impedance module must be greater than the last recorded value. The variation of the impedance module in the low frequency region indicates a constant increase in charge transfer resistance as the immersion time increases. The above is consistent with that observed in the analysis of the Nyquist diagram.

The observed behavior is consistent with that reported in other studies [39,40,41,42,43,44], that is, Ti and its alloys tend to develop on their surface a protective oxide (TiO_2_) composed of a bilayer oxide where the outer layer is porous and the inner layer is a barrier.

**Figure 7:**
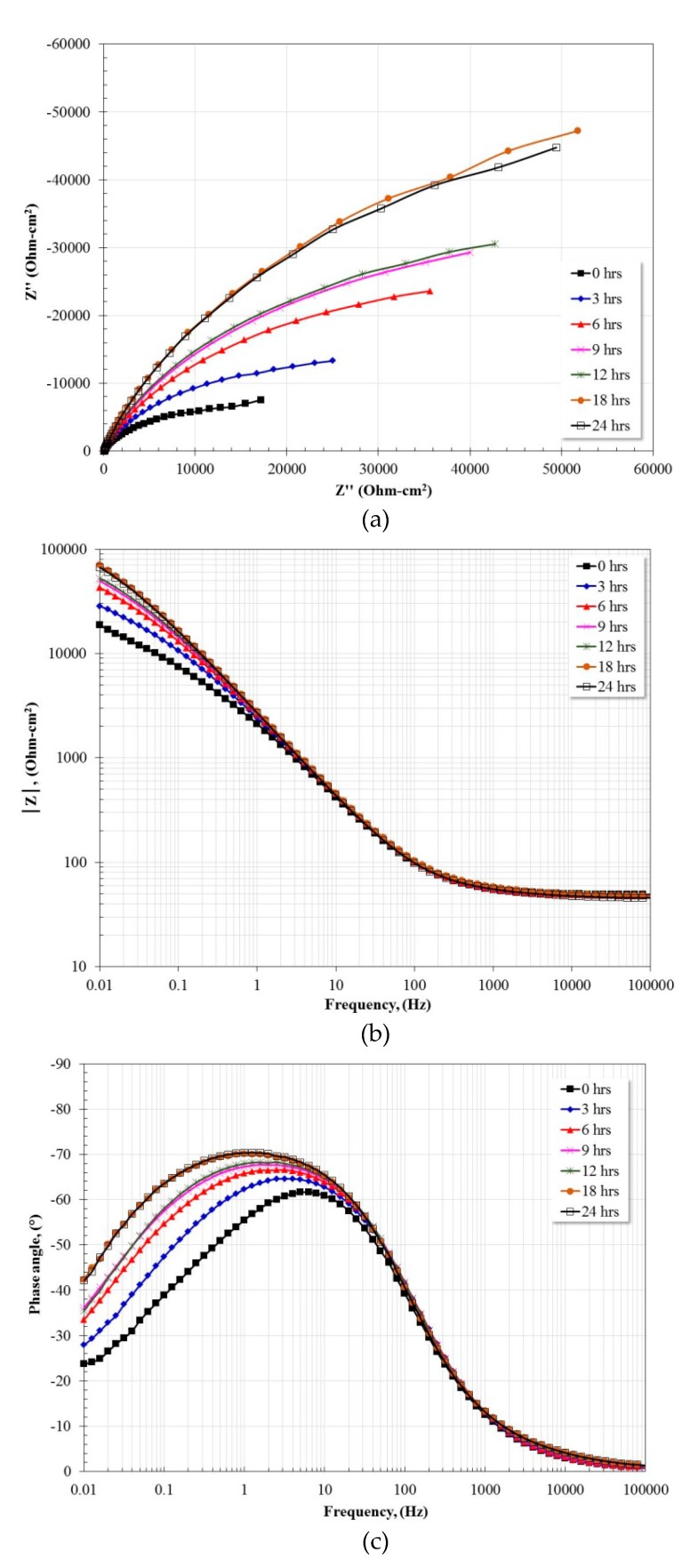
Nyquist (**a**) and Bode (**b**,**c**) plots for Fe40Al exposed in artificial saliva at 37 °C after 24 h.

Figure 7 shows the variation of the impedance spectra of the Fe40Al base intermetallic as a function of immersion time. The Nyquist diagram (Figure 7a) shows the apparent presence of a single capacitive semicircle whose diameter increases as a function of immersion time until 18 h. After 18 h, the diameter remains practically constant. This suggests the development of a stable protective layer. From Figure 7b, in the high frequency region, the presence of the corresponding plateau is observed, and, in the intermediate frequency region, a linear relationship between the frequency and the impedance module is observed with a constant increase in its slope. In the low frequency region, a constant increase in the impedance module is observed without defining the low frequency plateau formation. Figure 7c shows that the phase angle tends to zero at frequencies greater than 1000 Hz. In the intermediate frequency region a constant increase in the maximum phase angle of approximately 62° to 72° is observed. In addition, the broadening of the spectrum towards the low frequency region is visible, owing possibly to the presence of two overlapping time constants. This flattening behavior of the maximum phase angle as well as its broadening has been associated with the passivation of the metal surface [45]. The phase angle tends to zero at frequencies below 0.01 Hz. The above indicates a constant increase in charge transfer resistance as well as what was observed with the constant increase in the diameter of the capacitive semicircle.

**Figure 8:**
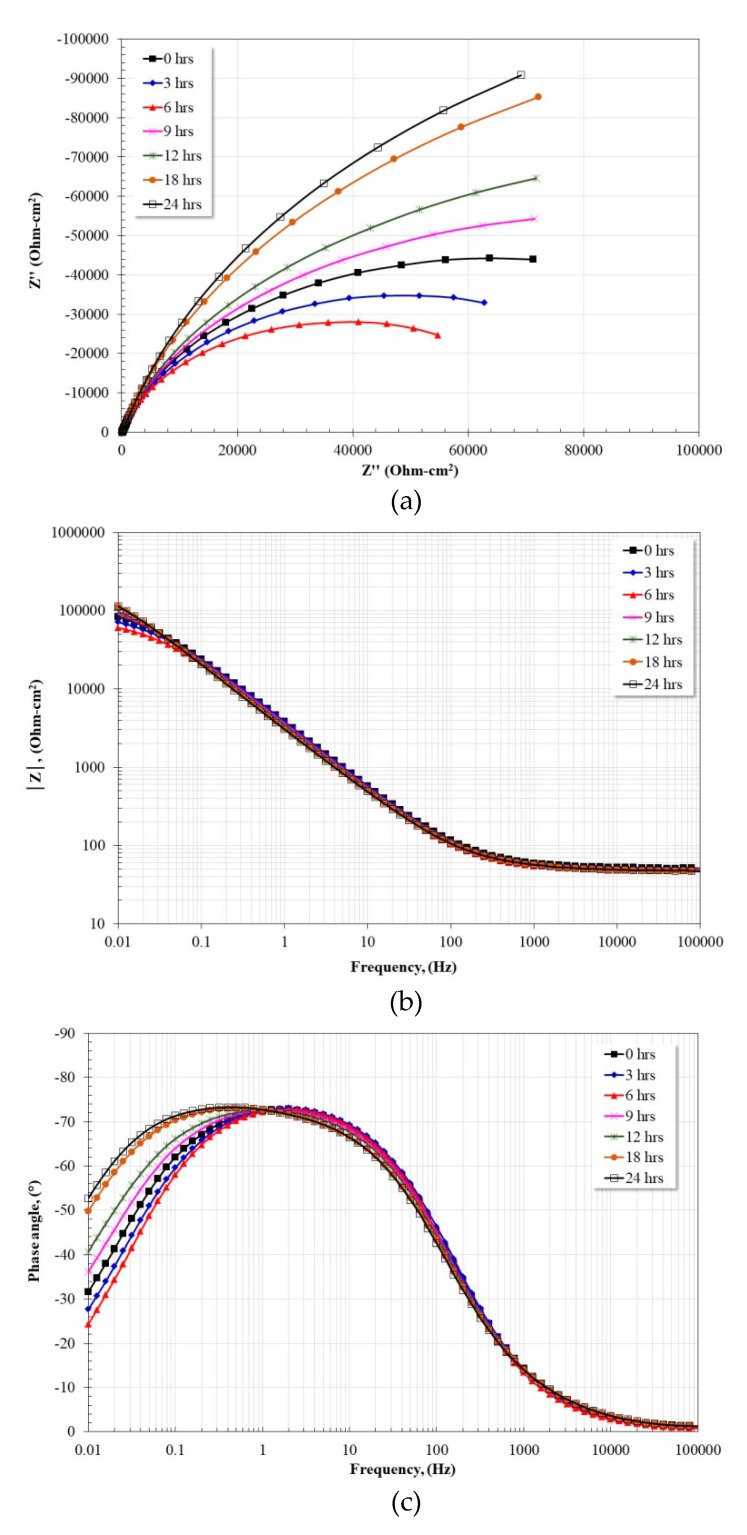
Nyquist (**a**) and Bode (**b**,**c**) plots for Fe40Al-3Ni exposed in artificial saliva at 37 °C after 24 h.

Figure 8 shows the evolution of the impedance spectra as a function of the immersion time of the Fe40Al-3Ni intermetallic. From the Nyquist diagram (Figure 8a) the apparent presence of a single capacitive semicircle is observed. At times less than 9 h, the diameter of the semicircle tends to decrease, and subsequently a constant growth is observed until the end of the test. The Bode diagram (Figure 8b) shows a constant behavior at frequencies greater than 0.1 Hz, namely the presence of the high frequency plateau and a practically constant log–*f*-log |*Z*| relationship. However, at lower frequencies the impedance module shows the same behavior observed in the Nyquist diagram, that is, a tendency to decrease at times less than nine hours, and a subsequent increase until the end of the test. The initial behavior observed may be associated with a greater dissolution of Fe and a subsequent enrichment of the protective film with NiO, which improved its corrosion resistance. From the Bode diagram (Figure 8c) at frequencies greater than 1 Hz a practically constant behavior is observed, and at lower frequencies the broadening and growth of the phase angle spectrum is evident. The shape and evolution of the phase angle spectrum is indicative of the presence of two overlapping time constants. The phase angle tends to zero at frequencies below 0.01 Hz.

**Figure 9:**
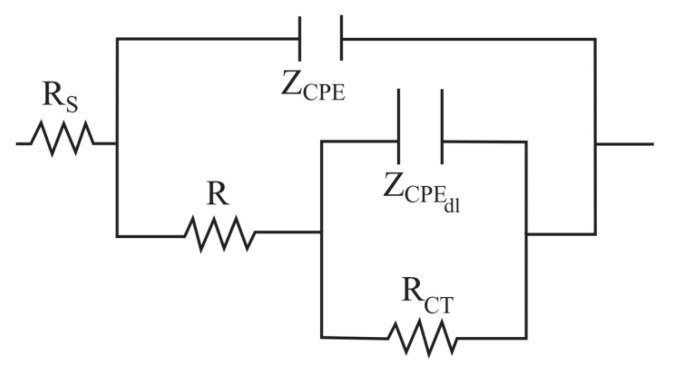
Equivalent circuit used to fit the impedance spectra.

According to all of the above, the impedance spectra can be adjusted according to the equivalent circuit of Figure 9.

The first time constant associated with the capacitive response of the work electrode surface is represented by R and Z_CPE_, where R is the resistance and Z_CPE_ is the impedance of the constant phase element (CPE). The second time constant associated with the double electrochemical layer is represented by R_CT_ and Z_CPEdl_, where R_CT_ is the charge transfer resistance and Z_CPEdl_ is the impedance of the CPE and Rs is the resistance of the electrolyte. In this model the capacitances (C) have been replaced by the CPE, which is commonly used to compensate the irregularities in the surface of the working electrode [46]:

(1) $$Z_{\mathrm{CPE}}=\left(\frac{1}{Y_{0}}\right){\left(j\omega \right)}^{-n}$$

where; Y_0_ = magnitude of the CPE (Ω^−1^ cm^−2^ s^n^), ω = angular frequency (rad s^−1^), j^2^ = −1 is the imaginary number and *n* = surface heterogeneity. The values of *n* vary from 0 < *n* <1. In general, if *n* = 1, CPE = C; if *n* = 0.5, CPE = Z_W_ (Warburg impedance); if *n* = 0, CPE = R. Based on the above, the capacitance can be calculated according to [46]:

(2) $$C_{\mathrm{CPE}}=\frac{{\left(Y_{0}R\right)}^{1/n}}{R}$$

Table 4 shows the fitting values of the impedance spectra of all the materials evaluated at 24 h of immersion.

From the analysis of the different electrochemical parameters, it is observed that the properties of the oxide developed on all the alloys (R, C) are within the same order of magnitude. The differences observed may be due to the thickness and heterogeneity of each oxide. In general, it is observed that the oxide developed onto Ti showed the lowest resistance and capacitance value and the highest *n* value. The above may be associated with the development of a thin oxide with excellent capacitive properties and lower surface heterogeneity. On the other hand, the oxide developed onto intermetallic alloys showed slightly higher resistance values and similar capacitance values. The similarity in the capacitance values is congruent because the main component of the protective oxide is Al_2_O_3_ and the presence of the third alloy element contributed to the formation of an oxide with different thickness and/or different surface defects. On the other hand, according to the R_CT_ and C_dl_ values, it is observed that the R_CT_ values coincide in magnitude with those polarization resistance values shown in Figure 4; the C_dl_ values are also very similar. This suggests that Al_2_O_3_-based oxides show capacitive properties similar to TiO_2_, and the differences observed may be associated with the surface defects of the oxides developed on the surface of the alloys.

**Figure 10:**
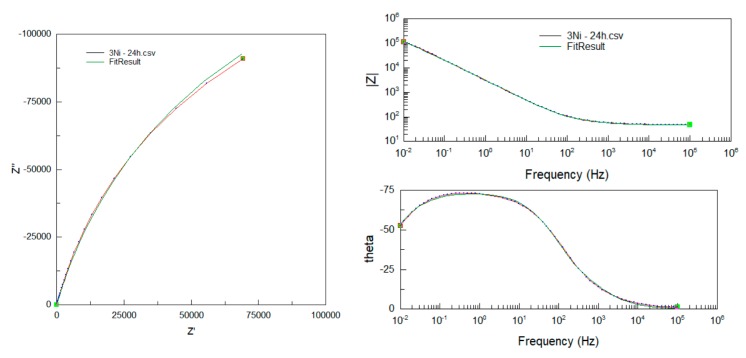
Adjustment of the experimental data of the Fe40Al-3Ni intermetallic with the proposed equivalent circuit.

As an example, Figure 10 shows the adjustment of the experimental data with respect to the proposed equivalent circuit (Figure 9) for the case of the Fe40Al-3Ni intermetallic.

The degradation of the materials depends largely on the chemical composition of the electrolyte in which it is immersed. It has been recognized that the ions present can be categorized into two classes according to the properties of their surrounding hydration shield [47], namely, water-structure-making anions, or cosmotrope anions, ($SO_{4}^{2-}$, $HPO_{4}^{2-}$, $CrO_{4}^{2-}$, *F*^−^, $HCO_{2}^{-}$, $CH_{3}SO_{2}^{-}$) and water-structure-breaking anions, or chaotrope anions, (*Cl*^−^, *Br*^−^, *I*^−^, $ClO_{4}^{-}$, $NO_{3}^{-}$, *SCN*^−^). The chemical composition of artificial saliva used in this study reflects the presence of both types of anions; however, only chaotropic anions are capable of initiating a pitting corrosion process, and the cosmotropic anions break the passive film only at high values of potential.

Notwithstanding the foregoing, the commonly accepted degradation mechanisms are based on the formation and dissolution reactions of the metal oxides considering a uniform corrosion process that generally occurs in conditions close to the rest potential.

It is known that in conditions close to those of resting potential, iron aluminides show a rapid tendency to passivation due to the establishment of a film of Al (III) oxide/hydroxide onto surface. However, its corrosion resistance can be affected by the presence of iron oxides, therefore, the addition of a third element improves the corrosion resistance due to the co-existence of their respective oxides together with Al_2_O_3_ [22,26,27,28,29].

The electrochemical reactions associated with the metal solution of the base intermetallic (Fe40Al) are [26,27]:

(3) $$\mathrm{FeAl}↔{\mathrm{Fe}}^{3+}+{\mathrm{Al}}^{3+}+6e^{-},$$

(4) $$6H_{2}O+6e^{-}↔3H_{2}+6{\mathrm{OH}}^{-},$$

The anodic reaction causes a superficial film of Al-Fe oxy-hydroxides which quickly becomes Al_2_O_3_:

(5) $$2{\mathrm{Al}}^{3+}+3H_{2}O↔{\mathrm{Al}}_{2}O_{3}+6H^{+}$$

In this case, the iron cations remain in solution and the Al-rich layer will provide protection to the intermetallic. However, the possible reduction of iron ions into the Al_2_O_3_ film will cause the presence of porosity affecting its corrosion resistance [26,27]:

(6) $$2{\mathrm{Fe}}^{3+}+2H_{2}O↔Fe_{2}O_{3}+6H^{+},$$

Despite the high values of polarization resistance (Figure 4) and charge transfer resistance (Figure 5) shown by all the intermetallic materials evaluated, the intermetallic alloy modified with Co addition showed the lowest performance.

According to reported studies [45,48,49], in acid and neutral solutions, Co shows an active dissolution behavior forming cobaltous ions (Co^2+^) according to the following reaction:

(7) $$\mathrm{Co}\to {\mathrm{Co}}^{2+}+2e^{-},$$

Therefore, except for the behavior exhibited with the addition of Co, the other ternary alloys showed an improvement in the corrosion resistance of the base intermetallic (Fe40Al). The above is due to the simultaneous formation of the oxide of the third element added, which possibly favored the formation of a layer of Al_2_O_3_ with fewer internal defects, which in turn reduced the continuous dissolution of the Fe. This improvement in corrosion resistance was greater with the addition of Ni and Ti.

## 4. Conclusions

Based on the different studies carried out on the intermetallic alloys evaluated, the following conclusions can be established.

The addition of a third alloy element (Cr, Co, Ni, Ti) does not alter the crystal structure of the binary intermetallic alloy.

In short-term corrosion tests (polarization curves) the results obtained showed that all Fe40Al-3X alloys exhibited a dual active-passive behavior. The addition of a third alloy element caused a decrease in the corrosion rate of the base intermetallic. With the addition of Ti the best performance was obtained, its icorr values were similar to those of Ti. This behavior could be associated to the modification of the Al-oxide by TiO_2_. All the ternary Fe40Al-3X alloys presented minor corrosion rates than other ternary intermetallic alloys.

In longer corrosion tests (OCP, LPR and EIS measurements), the Fe40Al-3Ni alloy showed the best performance of all the materials evaluated, followed by the Fe40Al-3Ti alloy. In particular, the LPR measurements showed very similar results to those observed with the polarization curve tests, that is, the modification of the intermetallic Fe40Al with the addition of Ni or Ti, substantially improved its corrosion resistance. In addition, according to the EIS measurements, it was observed that all the materials evaluated show similar electrochemical impedance spectra; the above suggests that the corrosion and protection mechanisms are similar. The results obtained suggest that the addition of a third alloy element causes the formation of an Al_2_O_3_ layer with fewer internal defects, which in turn reduced the continuous dissolution of Fe. The addition of cobalt does not improve the corrosion resistance of the intermetallic base due to its preferential dissolution.

The modeling of the impedance spectra suggests that, under the experimental conditions evaluated here, the oxides based on Al_2_O_3_ show capacitive properties similar to TiO_2_.

## Figures and Tables

**Table 1:** Chemical composition of the synthetic saliva (pH = 6.5).

Compound	Concentration (g/L)
NaCl	0.600
KCl	0.720
KH_2_PO_4_	0.680
Na_2_HPO_4_·12H_2_O	0.856
KSCN	0.060
CaCl_2_·2H_2_O	0.220
KHCO_3_	1.500
Citric acid	0.030

**Table 2:** Electrochemical parameters of the Fe40Al based alloys tested in artificial saliva at room temperature.

Alloy	Ecorr (mV)	Ba (mV/Dec)	Bc (mV/Dec)	i_corr_ (mA/cm^2^)	Corrosion Rate (mm/yr)	E_pit_ (mV)	I_pit_ (mV)
Fe40Al	−571	600	244	0.0021	0.0236	−166.5	0.008
Fe40Al-3Cr	−445	696	197	0.0015	0.0171	−155	0.0022
Fe40Al-3Ti	−582	162	110	0.00058	0.0064	−68.2	0.004
Fe40Al-3Co	−592	382	262	0.0016	0.01846	-	.
Fe40Al-3Ni	−507	625	173	0.0017	0.019	−162.6	0.0028
Titanium	−488	320	168	0.00054	0.0047	-	-

**Table 3:** Corrosion rates of selected dental alloys.

Alloy (wt.%)	Corrosion Rate (mm/yr)	Reference
Ti-6Al-4V	0.011	[34]
67Ni-14Cr-8Mo-7.5Ga	0.00024	[32]
Ti-6Al-7Nb	0.00031	[35]
Ti-13Nb-13Zr	0.00033	[35]
TiNi	0.00033	[33]
Ti-5Al-2.5Fe	0.00047	[33]
Ti-4.5Al-3V-2Mo-2Fe	0.00084	[33]
63Ni-22Cr-9Mo	0.00086	[32]
Ti-5Al-3Mo-4Zr	0.00108	[33]
74.5Ni-11.5Cr-3.5Mo	0.00139	[32]
68.5Ni-15.5Cr-14Mo1Al	0.00196	[32]
Ti-6Al-4V	0.00208	[33]
76.9Ni-12.6Cr-5Mo-2.9Al-2Be	0.046	[32]
80.7Ni-13.3Cr-4V-1.8Be	0.0629	[32]
75.7Au-4.7Pd–11.1Ag–8.4Cu–1.4Zn (Ney-Oro-B2)	0.502	[31,36]
Zr5Ti, Zr25Ti, Zr45Ti	0.0078-0.0229	[37]

**Table 4:** Electrochemical parameters of the electrochemical impedance spectroscopy (EIS) spectra after 24 h of immersion in artificial saliva.

Alloy	Rs (Ω·cm^2^)	R (Ω·cm^2^)	Yo (Ω^−1^·cm^−2^·s^n^)	n	C (μF·cm^−2^)	R_CT_ (Ω·cm^2^)	Yo_dl_ (Ω^−1^·cm^−2^·s^n^)	n_dl_	C_dl_ (μF·cm^−2^)
Ti	47.45	21.94	6.70 × 10^−6^	0.98	5.60 × 10^−6^	94,488	3.14 × 10^−5^	0.80	4.12 × 10^−5^
Fe40Al	45.69	42.55	6.04 × 10^−5^	0.76	9.19 × 10^−6^	120,390	2.63 × 10^−5^	0.86	3.17 × 10^−5^
Fe40Al-3Co	45.38	240.9	4.12 × 10^−5^	0.80	1.30 × 10^−5^	77,669	4.41 × 10^−5^	0.78	6.24 × 10^−5^
Fe40Al-3Cr	49.32	24.38	1.10 × 10^−5^	0.86	7.81 × 10^−6^	80,720	6.99 × 10^−5^	0.76	1.21 × 10^−4^
Fe40Al-3Ni	48.01	68.93	3.83 × 10^−5^	0.83	1.14 × 10^−5^	314,180	3.25 × 10^−5^	0.82	5.41 × 10^−5^
Fe40Al-3Ti	48.21	33.53	3.59 × 10^−5^	0.86	1.20 × 10^−5^	188,530	4.82 × 10^−5^	0.80	8.37 × 10^−5^
